# Time-to-pregnancy and pregnancy outcomes in a South African population

**DOI:** 10.1186/1471-2458-10-565

**Published:** 2010-09-21

**Authors:** Braimoh Bello, Danuta Kielkowski, Dick Heederik, Kerry Wilson

**Affiliations:** 1 Reproductive Health and HIV Research Unit, University of the Witwatersrand, Johannesburg, South Africa; 2 Division of Environmental Epidemiology, Institute for Risk Assessment Sciences, Utrecht University, Netherlands; 3 Epidemiology and Surveillance Unit, National Institute for Occupational Health, Johannesburg, South Africa; 4 School of Public Health, University of the Witwatersrand, Johannesburg, South Africa

## Abstract

**Background:**

Time-to-pregnancy (TTP) has never been studied in an African setting and there are no data on the rates of adverse pregnancy outcomes in South Africa. The study objectives were to measure TTP and the rates of adverse pregnancy outcomes in South Africa, and to determine the reliability of the questionnaire tool.

**Methods:**

The study was cross-sectional and applied systematic stratified sampling to obtain a representative sample of reproductive age women for a South African population. Data on socio-demographic, work, health and reproductive variables were collected on 1121 women using a standardized questionnaire. A small number (n = 73) of randomly selected questionnaires was repeated to determine reliability of the questionnaire. Data was described using simple summary statistics while Kappa and intra-class correlation statistics were calculated for reliability.

**Results:**

Of the 1121 women, 47 (4.2%) had never been pregnant. Mean gravidity was 2.3 while mean parity was 2.0 There were a total of 2467 pregnancies; most (87%) resulted in live births, 9.5% in spontaneous abortion and 2.2% in still births. The proportion of planned pregnancies was 39% and the median TTP was 6 months. The reliability of the questionnaire for TTP data was good; 63% for all participants and 97% when censored at 14 months. Overall reliability of reporting adverse pregnancy outcomes was very high, ranging from 90 - 98% for most outcomes.

**Conclusion:**

This is the first comprehensive population-based reproductive health study in South Africa, to describe the biologic fertility of the population, and provides rates for planned pregnancies and adverse pregnancy outcomes. The reliability of the study questionnaire was substantial, with most outcomes within 70 - 100% reliability index. The study provides important public information for health practitioners and researchers in reproductive health. It also highlights the need for public health intervention programmes and epidemiological research on biologic fertility and adverse pregnancy outcomes in the population.

## Background

Research into reproductive health outcomes in South Africa is limited and some areas are under-researched [1]. The majority of reproductive health research carried out in the country has been in clinical settings or selected populations. Currently, there are no studies on the distribution of fecundity (biologic fertility), the prevalence of clinical infertility and many adverse pregnancy outcomes for a representative South African population. Without these rates, it is impossible to discuss patterns, trends or causal relationships linking exposures to reproductive health outcomes. This makes it difficult to develop evidence-based health policies which are a core component of primary prevention of diseases.

The lack of epidemiological reproductive studies in South Africa, and many other African countries, is largely because these studies can be complex and difficult to conduct. Some hurdles in conducting these studies lie in cultural beliefs and practices around reproductive health issues. Other obstacles include the need for appropriate measurement tool, proper study design to minimize bias and confounding, difficulty in recruiting participants and the need for a large sample size as some reproductive health outcomes are rare [2].

Information on reproductive health outcomes, including time-to-pregnancy (TTP) and pregnancy outcomes, can be readily collected by means of a questionnaire tool [3,4]. The use of questionnaires makes large-scale reproductive health studies feasible in under-resourced settings, as valid information on outcomes and exposures can be collected without resource-intensive clinical or laboratory investigations [5]. However, there are limitations to collecting such information retrospectively by means of a questionnaire. These limitations could introduce different forms of bias, such as bias due to pregnancy recognition and poor recall, into the study [3,6]. Adverse outcomes like spontaneous abortions may be underreported due to late recognition of pregnancy and reluctance to recall unpleasant events. However, most of these problems can be managed if the studies are appropriately designed, conducted and analyzed. A good starting point would be to develop or adapt a reliable tool that is validated in the population setting.

Although questionnaires measuring TTP and other reproductive outcomes have been validated in many Western settings [7-10], there were reasons to be concerned about how well these outcomes can be measured in an African setting. The reasons included low levels of education and cultural beliefs which may lead to poor recall of information. Also, social desirability may cause incorrect reporting of measures like contraception, planned pregnancy and fertility. Our study objectives were to describe TTP, determine the prevalence of adverse pregnancy outcomes in a representative South African population and assess the reliability of the questionnaire tool used for the study. This is the first study in South Africa to do so.

## Methods

### Study population

The study was carried out in Potchefstroom, a small urban town in the North-West Province of South Africa with an approximate population of 128 000 people [11]. It is a university town and also home to industries such as engineering and agriculture. Potchefstroom Municipality was chosen for the study because its relative population structure was similar to the general South African population [11].

The inclusion criteria for the study were: being of reproductive age [18 - 49 years], residing in the community and having been pregnant or tried to conceive. The study was cross-sectional with the sample designed to be representative of the population. The sampling frame used was South Africa's most recent census [11] which gave information on population distribution by gender, age, race and voting wards. The calculated sample size was 1079 women, based on estimates from a previous pilot study [12]. The sample size was increased slightly for coloreds (mixed race) and Asians to allow for representation of these minority subgroups. The South African Demarcation Board provided a map of ward boundaries and a list of streets in all 21 wards. The municipal authorities showed interest in reproductive health information by ward, so we did not conduct cluster sampling; rather we sampled from all wards. The number of women sampled from each race group and each ward was proportional to the number of eligible women in that race group and ward. We exceeded our estimated sample size and collected data on 1121 women (table 1).

**Table 1:** Population distribution of reproductive age women in Potchefstroom, estimated sample size and actual data collected

*Population group*	*Population* *Size*	*Estimated* *Sample size*	*Data collected*
Black	26, 710 (69%)	754 (70%)	709 (63%)
White	9,127 (24%)	252 (23%)	241 (21%)
Colored	2,500 (6.5%)	69 (6.4)	140 (13%)
Asian	145 (0.4)	4 (0.4)	31 (3%)

Total	38, 482	1079	1121

The sampling units for the study were housing units. There was approximately one eligible woman per housing unit in the population. We had information on the total number of housing units per ward and took a systematic sample of households from a random starting point within each ward. A sampling interval was calculated for each ward; wards had different sampling intervals due to differing population structures. All women of reproductive age were selected per household. If a woman refused to participate in the study or if after two visits (day and evening), no interview could be conducted in the selected household, the immediate household to the right was approached.

### Study questionnaire

The study retrospectively ascertained information using a questionnaire. We adapted the European Study Group reproductive health questionnaire [13] to our setting and objectives, and piloted it in the community in 2006 [12]. The questionnaire was translated and back translated from English to Setswana and Afrikaans, the two other major languages spoken in the community. The questionnaire sought information on current socio-demographic variables, environmental and occupational exposures, summary of pregnancy history, contraceptive use, TTP and pregnancy outcomes. Detailed information on TTP and pregnancy outcomes was sought for the most recent pregnancy only. For women who had been pregnant, TTP data was defined for those who planned their most recent pregnancies: TTP was recorded as the number of months it took for a couple to conceive. Unplanned and accidental pregnancies where excluded. Among women who had never been pregnant but where trying, TTP was recorded as the waiting time from the starting time of attempt to the date of this survey. Pregnancy outcomes included spontaneous abortion, stillbirth, livebirth, induced abortion, pre-term birth and malformation at birth. For TTP, exposure history at the time of pregnancy attempt was sought. For pregnancy outcomes, exposure history during pregnancy was sought.

### Data collection

Experienced community interviewers who carried out the pilot study were trained further to conduct interviews for this study. Data was collected from August 2007 to March 2008. Interviewers were closely supervised and questionnaires were checked for quality and completeness. Interviewers' awareness of a planned random repeat study also enhanced data quality. Each interviewer, assigned to specific wards, visited selected households and conducted face-to-face interviews with eligible women. Quality control procedures were followed throughout the study and all data was confidential. Written informed consent was obtained from participants. The Human Research Ethics Committee of the University of the Witwatersrand approved the study.

Data collection was completed without major obstacles in the black and colored wards, according to the sampling scheme. Conversely, data collection was problematic in the white and Asian wards; there were difficulties in accessing many residential houses. Most women were rarely at home during working hours and the few eligible women approached often declined consent. Attempts to interview the women on weekends were unsuccessful. As a result, only a few interviews could be conducted for white and Asian women according to the sampling scheme. We then contacted major employers for permission to approach female staff at their workplaces for the study. The majority of the questionnaires on white and Asian women were collected this way.

A repeat study on a small number of the study participants was carried out to estimate questionnaire reliability. Women were randomly selected for re-interview a few weeks after their original interview. Data collection for the repeat study was completed in May 2008. Although we aimed to interview 112 (10%) of the women included in the main study we could only complete 73 (6.5%) interviews due to costs and logistical reasons. Ninety percent of reliability interviews were done face-to-face while the remaining 10% were done telephonically.

### Statistical analysis

Data was captured twice in Epi-info (CDC, Atlanta, USA) and statistical analyses were carried out in Stata 10 (StataCorp, 2008, Texas, USA). Data was summarized using means and proportions. For reliability analysis, intra-class correlations (ICC) were calculated for continuous variables, while observed agreement and kappa statistics were calculated for categorical variables. In addition, differences between TTP values for the main and repeat interviews were calculated. Odds ratios (OR) were computed for potential determinants of TTP reliability. Analyses were done at an alpha level of 0.05 and 95% confidence interval (CI) were reported for OR and ICC.

## Results

### Main study

The response rate for the black and colored communities was over 95%; hence we did not collect information on non-response. For white and Asian women, there was very good participation at their workplaces with the response rate over 90%. Overall, the response rate for the study was quite high except that there were difficulties in ascertaining how representative the data from the white and Asian wards were.

#### Socio-demographic characteristics

Table 2 shows the main demographic characteristics of study participants. The majority (69%) of participants were between 20 and 40 years of age during the study. Half of the most recent pregnancies occurred in the age group of 20 to 29 years. Similar proportions of women reported working currently and at most recent pregnancy. A worrying proportion (17%) of participants reported smoking during their most recent pregnancy. Most women (97%) achieved primary education. Social group followed an expected pattern based on South African distribution of wealth: many of the black and colored participants were expected to be of a lower social-economic group (data not shown).

**Table 2:** Socio-demographic characteristics of study participants

*Variables*	*n (%)*
Current age	
< 20	22 (2.0)
20- 29	362 (32.4)
30 - 39	407 (36.4)
≥ 40	326 (29.2)
Age at most recent pregnancy*	
< 20	131 (12.2)
20- 29	563 (52.5)
30 - 39	349 (32.6)
≥ 40	29 (2.7)
Current Education	
None	35 (3.1)
Primary	205 (18.3)
Secondary	667 (59.5)
Tertiary	214 (19.1)
Social group by income	
Low	686 (61.9)
Middle	245 (22.1)
High	177 (16.0)
Employment	
Currently	419 (37.4)
During pregnancy*	449 (41.9)
Smoking	
Ever smoked cigarettes	190 (17.0)
Ever used snuff	202 (18.1)
Smoked during recent pregnancy*	182 (17.0)
Alcohol consumption during recent pregnancy*	
Never	867 (85.3)
Sometimes	116 (11.4)
Usually	33 (3.2)

Data is presented for all participants (n = 1121) except for variables* at most recent pregnancy (n = 1074)

#### TTP and adverse Pregnancy outcomes

**Figure 1:**
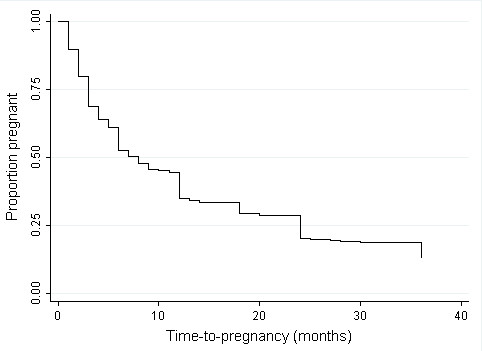
**Population time-to-pregnancy distribution censored at 36 months**. Most women (33%) achieved pregnancy in the first 3 months with a gradual tailing off to the right. The proportion of women who were pregnant after 6, 12 and 24 months was 50%, 68% and 83% respectively.

The total number of women who have been pregnant was 1074. Forty seven women (4.2%) failed to achieve pregnancy, of which 25 of them (53%) have tried for more than a year. There were a total of 2467 pregnancies; most (87%) resulted in live births, 235 (9.5%) in spontaneous abortion and 54 (2.2%) in still births. Of the women who have been pregnant, 174 (16%) experienced at least a spontaneous abortion and 45 (4.2%) experienced more than one. There was an average of two live births per woman while there were only four cases of induced abortions reported by three participants. Twenty percent of most recent pregnancies were preterm births and no malformation at birth was reported. Although 757 women (71%) used some form of contraceptive prior to pregnancy, the proportion of planned pregnancies was only 39%. Side effects were a common reason given for stopping contraceptives. The proportion of planned pregnancies differed significantly by race; whites (60%), blacks (31%), coloreds (41%) and Asians (39%) (*P *< 0.001). TTP data was available for 43% (39.4%, planned pregnancies and 4.2%, never-pregnant women) of participants. The TTP distribution (Figure 1) had a mode and median of 3 and 6 months respectively, with 68% achieving pregnancy in the first year.

### Questionnaire reliability

A total of 73 interviews were carried out for reliability analysis. All randomly selected participants consented to a repeat interview. The repeat interviews were conducted between one and four months after the main study. The median interval was 2.5 months.

#### Reliability of socio-demographic variables

Participants' current age was sought using date of birth. A reliability analysis on age showed an ICC of 0.99. There was 100% agreement for race. Agreement was equally excellent for current work status (90%) and current household income (87%).

#### Reliability of contraceptive use, planned pregnancy and time-to-pregnancy

**Figure 2:**
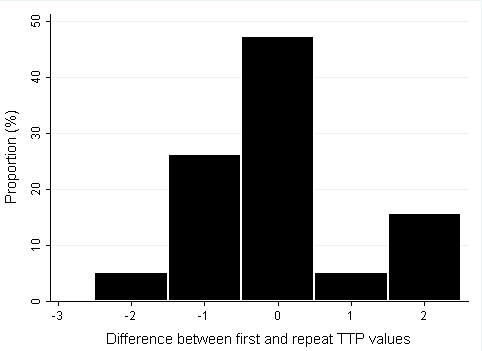
**The distribution of difference between main and repeat time-to-pregnancy values censored at 14 months**. There were no differences between first and repeat data for 48% of women and all women had a maximum of two months discrepancy.

The reliability for planned pregnancy was very high with an observed agreement of 90% and a kappa of 0.79 (p-value < 0.001). The observed agreement for contraceptive use was also high (76%) but the kappa was a slight 0.4 (P-value < 0.001). TTP data for the main and repeat interview were available for 22 participants. Reliability for TTP was quite good; the ICC for all 22 individuals was 0.63 (*P *< 0.001). The ICC further improved to 0.97 (n = 20, *P *< 0.001) and 0.96 (n = 21, *P *< 0.001) when TTP was censored at 14 months and at 24 months respectively. When TTP was categorized as 1 to 6 months, 7 to 12 months and more than 12 months, the observed agreement was 87% with a weighted kappa of 0.75 (*P *< 0.001). Further analysis on TTP reliability was done by analyzing the differences between the first and second values. For TTP values censored at 14 months, 48% of the data had no discrepancy while all data where within two months discrepancy (figure 2).

#### Determinants of TTP reliability

To determine factors that may affect TTP reliability, women were categorized as discrepant if there was a difference between their first and second TTP values, and non-discrepant if there was none. Fifty-five percent of women were discrepant. Although discrepancy varied by race, smoking, alcohol use and period of recall, only period of recall was statistically significant. Due to small numbers, a multivariable regression was not performed on the data (table 3).

**Table 3:** TTP reliability by important socio-demographic variables

*Determinant*	*Unadjusted odds ratio*	*95% CI*
Race		
White	1	
Black	2.33	0.34, 16.18
Current age (years)	0.92	0.76, 1.11
Age at pregnancy attempt (years)	0.69	0.48, 1.01
Ever smoked		
No	1	
Yes	1.78	0.13, 23.52
Smoked at pregnancy attempt		
No	1	
Yes	3	0.25, 35.33
Alcohol at pregnancy attempt		
No	1	
Yes	1.67	0.27, 10.33
Education at pregnancy attempt		
Less than High School	1	
High School or More	0.67	0.11, 3.92
Currently working		
No	1	
Yes	0.96	0.16, 5.64
Pregnancy outcomes		
Live births	1	
Pregnancy loss	1.56	0.12, 20.85
Period of recall		
<= 5 years	1	
> 5 years	21.33	1.8, 251.26
Chronic disease at attempt		
No	1	
Yes	1.78	0.13, 23.52

#### Reliability of pregnancy outcomes

Both induced abortions and birth defects showed 100% agreement as none of the outcomes were reported by the 73 repeat study participants. The reliability of the other pregnancy outcomes was equally excellent (table 4).

**Table 4:** Reliability of pregnancy outcomes

*Outcome*	*n*	*Measure of reliability*
No of pregnancies^a^	73	0.98 (0.97 - 0.99)
No of live births^a^	73	0.95 (0.92 - 0.97)
No of spontaneous abortions^a^	73	0.91 (0.88 - 0.95)
No of still births^a^	73	0.41 (0.21 - 0.60)
No of pregnancy losses^a ^(spontaneous abortion or stillbirth)	73	0.96 (0.94 - 0.98)
Outcome of most recent pregnancy^b^ (livebirth, stillbirth, spontaneous abortion, Induced abortion)	63	0.96 (kappa 0.6)**
Outcome of most recent pregnancy^b^ (preterm, term, delayed)	65	0.98 (kappa 0.9)**

^a^Intra-class correlation reported for first five measures

^b^Agreement and weighted kappa reported for outcomes of pregnancy

## Discussion

Although sexual health, contraception and demographic fertility have been extensively studied in South Africa, this is the first study to describe fecundity distribution and rates of adverse pregnancy outcomes for a representative South African population. The findings from this study can be extrapolated to the South African general population because the relative racial composition of the study population is similar to that of the general population. The fertility findings from our study are in line with an average of two children per woman that has been previously reported for South Africa [14,15]. The data shows that the rates of spontaneous abortion in South Africa seemed fairly high. Although there are no South African population data to compare these rates to, the proportion of women who experienced spontaneous abortion (16%) is similar to the 11% reported in a clinic-based study of 1920 women conducted in two South African hospitals [16]. In an occupational study carried out in Johannesburg, the rate of spontaneous abortion was 19% for women exposed to ethylene oxide and 10% in those unexposed [17]. Also, our rate is strikingly similar to what has been reported for some European countries, for example 15% for Denmark, 16.5% for Germany and 17.4% for Poland [18]. Contrary to expectations, this comparability suggests that there may be little or no under-reporting of pregnancy loss in this population.

The proportion of women who used contraceptives before their most recent pregnancies (71%) is similar to the 66% of women who reported currently using contraceptive in the 2003 South African demographic and health survey (SADHS) [19]. Although contraceptive use prior to pregnancy is fairly high (71%), only about half of those women stop their contraceptive to achieve pregnancy resulting in the low proportion of planned pregnancies observed (39%). The common reason indicated for stopping contraceptives was side effects. Previous studies in South Africa and elsewhere have also reported high frequency of contraceptives discontinuation due to side effects [20-22]. There is therefore a need for further studies to explore this observation with an aim to increasing planned pregnancies.

The proportion of planned pregnancies (39%) was low when compared to what is normally reported for Western countries [23,24], but similar to the 42% obtained for the pilot study [12] and the 50% reported in the 2003 SADHS [19]. An earlier South African study also reported a high prevalence of contraceptive use, but a low proportion of planned pregnancies in rural South Africa [25]. Baird, et al. [3] noted that the proportion of planned pregnancies can be low even in populations where contraceptive use is high. Low proportions of planned pregnancy have also been reported for some European countries: 37% in Poland [18] and 41% in East Germany [13]. The observation that the rate of planned pregnancies was related to race (highest in white women and lowest in black women) has been reported in the SADHS [19] and in studies done in the US [26,27]. It is probable that the influence of race on pregnancy planning is through socio-economic (for example, education and income) disparities. From the view point of planning TTP studies, the low proportion of planned pregnancies has implications for sample size calculation and allocation of resources. This should be taken into account when planning TTP studies in Africa, especially outside of white communities. It is interesting that although the proportion of planned pregnancy is low, the rate of induced abortion appears negligible. Perhaps this reflects a culture where although pregnancies are usually unplanned, they are wanted when they occur. It may also be due to reporting bias where abortions are considered socially unacceptable and thus may be underreported.

The TTP distribution showed that majority of women achieved pregnancy in the first three months, with a gradual tailing off of the distribution to the right. Since, TTP distribution has not been previously studied in Africa; there were no African studies to compare this distribution to. However, the 68% of South African women achieving pregnancy in the first year is within the 67 to 85% range reported for five European countries in a multi-country population study [18]. An article on global infertility trend, published in 2009, showed that the rate of infertility (the proportion of women who fail to achieve pregnancy after 1 year of trying) ranges between 20 and 30% for African settings in contrast to 6 - 10% for some western countries [28]. A common misconception is that subfertility and adverse pregnancy outcomes are not a concern in Africa. Our results show that the distribution of biologic fertility and the rates of adverse pregnancy outcomes are similar (may be worse) to what is obtainable for developed countries. This raises important public health questions for South Africa and many African settings. Are there policies and interventions to protect the reproductive health of women and the health of their unborn children? These findings highlight the need for more research into biologic fertility and adverse pregnancy outcomes in this population. The role of environmental and occupational exposures should be investigated, given the rapid industrialization of South Africa in the past few decades. Also the effect of HIV and antiretroviral therapy on these outcomes should be continually assessed.

The reliability of the study questionnaire indicates that the study results are accurate and reliable. The reliability results give insight into the knowledge and perception of African women on their reproductive health. The high reliability observed was unexpected and may, again, be explained by the high levels of functional literacy in the population (97% of women attained primary education, a level usually associated with literacy), the quality of the questionnaire tool and the quality of training the study interviewers received. As expected, the reliability of demographic variables was excellent. There are four major race groups in South Africa - blacks, whites, Asians and coloreds. It is probable that some people may not always put themselves in the same group depending on context. Race is an important demographic variable in the country as it is highly correlated with income, education, culture and employment.

The high reliability observed for TTP data shows that TTP can be measured in an African setting by means of a questionnaire. The good reliability appears to be validated by the TTP distribution which is similar to what has been reported for other settings. A study measuring the reliability of TTP values of 70 women showed that TTP can be reproduced with two months accuracy in 95% of cases [9]. Another reliability study showed that about 45% of women (48% in our study) reported the same figure in the primary and repeat data [8]. We obtained similar but slightly better reliability than these studies. Of note is that reliability increased when TTP data was censored at 14 months and 24 months. Other studies have also shown that as TTP gets longer, accuracy tend to decrease [7,8,10]. This supports the analysis approach of censoring TTP data (especially in non-pregnancy based population studies) at a defined time, say 14 months. This fortunately does not reduce statistical power as most TTP data are in the first few months and biases (for instance time-trend bias) that may affect TTP do not usually operate in the first few months. Another analysis approach is to categorize TTP data. When we categorized TTP into 0 to 6 months, 7 to 12 months and greater than 12 months, we observed a high agreement of 87% and a weighted kappa of 0.75. The only variable which was significantly associated with TTP reliability was period of recall. Although recall of up to 20 years have been validated in Europe [4,29], some studies have restricted their recall period to shorter durations [13,18,30,31]. Our results underscore the need to reduce the period of recall and focus on the most recent pregnancies when collecting TTP data retrospectively; one is more likely to obtain accurate and reliable information for most the recent pregnancy.

The reliability of pregnancy outcomes was generally excellent. The low reliability observed for number of stillbirths is related to the differentiation of spontaneous abortion from stillbirth - we used 24 weeks as cut off in this study. When spontaneous abortion and stillbirth were grouped as pregnancy loss, the reliability increased considerably. Although there is scientific basis for distinction between both outcomes, some participants may not see this distinction as important. Interviewers should be trained to appreciate the distinction between both outcomes and to be patient with participants as they try to recall events and durations.

The findings of this study are useful for informing intervention, stimulating new epidemiological research in reproductive health, sample size calculations and planning for similar studies. The data is useful as reference when evaluating the effect of specific exposures on reproductive outcomes where an internal comparison group cannot be studied. Also, the study helps to introduce the concept of TTP as a measure of biologic fertility to the African context. Time-to-pregnancy, the number of non-contraceptive menstrual cycles it takes a couple to conceive, is an important reproductive health measure for a number of reasons. It is a validated and sensitive measure of fertility; its distribution indicates the biologic fertility of a population (3, 4). Also, it is a functional outcome of reproductive mechanisms in both men and women. Lastly, exposures that prolong TTP may cause other adverse reproductive outcomes (4, 5). TTP is useful in the fields of population, occupational and clinical reproductive health. The questionnaire tool used in this study can be used in occupational reproductive health research to study the effect of specific occupational exposures on TTP and pregnancy outcomes. We currently plan to use the tool to assess the effect of workplace exposures on fertility and pregnancy outcomes amongst laboratory workers and domestic workers in South Africa. Also, researchers in clinical reproductive health can use the tool to evaluate the effects of HIV infection and specific antiretroviral regiments on these outcomes.

## Conclusions

This is the first comprehensive population-based reproductive health study in South Africa. It describes the biologic fertility of the population and provides rates for planned pregnancies and adverse pregnancy outcomes. The median time-to-pregnancy in the population was 6 months, with 68% of women achieving pregnancy in the first year. About 10% of all pregnancies recorded resulted in spontaneous abortion while 20% of the most recent pregnancies were preterm births. The reliability of the study questionnaire was substantial, with most outcomes within 70 - 100% reliability index. The study provides important information for policy makers, practitioners and researchers in reproductive health in South Africa and similar settings.

## Competing interests

The authors declare that they have no competing interests.

## Authors' contributions

BB: Substantial contribution to conception and design, field work, literature review, analysis and interpretation of data, drafting and critically revising the article for important intellectual contribution and final approval of the version to be published. DK: Substantial contribution to conception and design, fieldwork and interpretation of data. Drafting and critically revising the article for important intellectual contribution and final approval of the version to be published. DH: Substantial contribution to literature review, analysis and interpretation of data. Drafting and critically revising the article for important intellectual contribution and final approval of the version to be published. KW: Substantial contribution to conception and design, fieldwork and interpretation of data. Drafting and critically revising the article for important intellectual contribution and final approval of the version to be published.

## Pre-publication history

The pre-publication history for this paper can be accessed here:

http://www.biomedcentral.com/1471-2458/10/565/prepub
